# Isolation of Indigenous Selenium Tolerant Yeast and Investigation of the Relationship Between Growth and Selenium Biotransformation

**DOI:** 10.15171/apb.2020.020

**Published:** 2019-12-11

**Authors:** Maryam Hosseindokht Khujin, Hamed Zare

**Affiliations:** ^1^Department of Genetics and Molecular Biology, School of Medicine, Isfahan University of Medical Sciences, Isfahan, Iran.; ^2^Cellular and Molecular Research Center, Birjand University of Medical Sciences, Birjand, Iran.; ^3^Pharmaceutical Biotechnology Department, School of Pharmacy, Shahid Beheshti University of Medical Sciences, Tehran, Iran.

**Keywords:** Yeast, Selenium, Biotransformation, Screening, *Saccharomyces cerevisiae*

## Abstract

***Purpose:*** Organic selenium compound such as selenomethionine plays a significant function in the response to oxidative stress. Saccharomyces cerevisiae have the ability to accumulate selenium and selenium biotransformation. Selection of indigenous selenium tolerant yeast is our goals. The relationship between cell growth and selenium biotransformation was also investigated.

***Methods:*** The screening of the yeast cell was carried out at two steps in order to select yeast with high capacity for resistance and accumulation of selenium. The isolates were selected according to produced high biomass at different concentrations of selenium. Secondly, best yeast strains from previous step were grown in presence of 25 mg/L of sodium selenite and organic selenium content was measured.

***Results:*** The S17 isolate showed had maximum organic selenium accumulation (2515 ppm) and biomass production (2.73 g/L) compared to the other isolates. The biomass production and organic selenium accumulation of the S17 during 120 hours was shown a direct relationship between growth and biotransformation.

***Conclusion:*** This increase in organic selenium content was achieved with yeast screening. It is interesting to know that organic selenium has high bioavailability and low toxicity compared with inorganic selenium. Therefore, finding yeast strains which are resistant to selenium can be very helpful in cancer prevention.

## Introduction

Selenium has been shown to be a crucial micronutrient of human diets.^1,2^ This element was discovered in 1817.^3,4^ selenium is located in glutathione peroxidase.^5,6^ This enzyme along with catalase and superoxide dismutase protects cells from the oxidation damage.^4,7^ It has been shown that selenium has important roles in prevention of several cancers such as prostate cancer.^6,8-10^ Deficiency of selenium is linked to the incidence of several diseases including heart disease, infertility, cancer and reversible cardiomyopathy that known as Keshan disease.^4,11,12^ A lot of people in many countries consume a small amount of selenium.^1^ Addition of inorganic salts of selenium in food products is one of the ways to overcome insufficiency of selenium, but this element has a narrow border for essential and toxic concentrations.^9,13^ Interestingly, organic selenium to be the most bioavailable for human consumption.^6,9^

*Saccharomyces cerevisiae* strains have the ability to absorb inorganic selenium and also they can transform inorganic selenium into organic selenium ingredients.^13,14^ The advantage of Seleno-yeast is easy ingestion, low cost and high content of selenomethionine.^1,10,15^ In this study, the ability of the yeast strains was measured in organic selenium production. In the following, the relationship between growth and organic selenium production was investigated.

## Materials and Methods

### Materials

Culture medium and salts were supplied by Merck (Germany). Master Mix PCR was obtained from Genaxxon. Standard yeast *S. cerevisiae* (PTCC 5157) was obtained from Iranian Biological Resource Center.

### Yeast isolates

The yeast isolates from our previous study^15^ were identified according to the criteria of Kurtzman and Fell.^16,17^ Molecular identification was done with PCR reaction.^18^ Lastly, the best yeast isolates with high resistance against selenium were confirmed by sequencing of the LSU rRNA gene D1/D2 domain of 26S rDNA and ITS region including 5.8S rRNA gene.^19-21^

### Screening for biomass production in the presence of selenium

The isolated strains were grown in SD (Sabouraud Dextrose) broth containing different concentrations of sodium selenite, i.e., 5 mg/L, 15 mg/L and 25 mg/L, and incubated at 30°C for 24 hours. The dry cell weight (DCW) was measured and strains with highest biomass were chosen for more screening.

### Screening for selenium biotransformation

Selected strains were grown in SD broth containing 25 mg/L of sodium selenite, and incubated at 30°C for 24 hours. The yeast cultures were centrifuged at 8000 g for 10 min, washed 3 times for elimination of selenium from the cell surface. Cell sediment was dried at 85°C and weighed. Total and inorganic selenium was determined according to the atomic absorption spectroscopy method.^22^ Calculation of organic selenium yield was obtained from the difference between the total and inorganic selenium.^22^

### Relationship between growth and selenium biotransformation

Two isolates with the high selenium tolerance and maximum organic selenium accumulation (S17 and S34) were chosen for more investigations. S17 isolate was grown in 5 ml SD broth and incubated at 30°C for 24 hours. One milliliters of this culture was added to 5 × 100 mL SD broth containing 25 mg/L of sodium selenite and was incubated at 30°C for 120 hours. Biomass production and organic selenium accumulation were measured at 24, 48 h, 72, 96 and 120 hours.

## Results and Discussion

### Screening for biomass production in the presence of selenium

**Figure 1:**
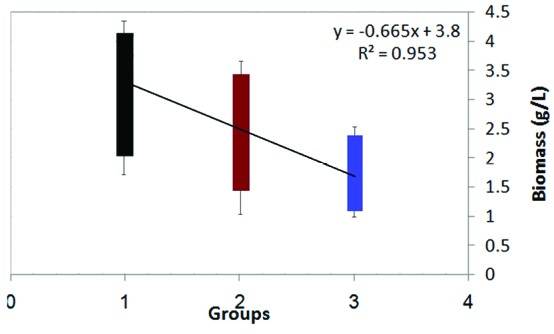
Biomass production in *Saccharomyces cerevisiae* isolates under different concentrations of selenium. Selenium concentration in group 1, group 2 and group 3 is 5, 15 and 25 mg/L respectively.

**Figure 2:**
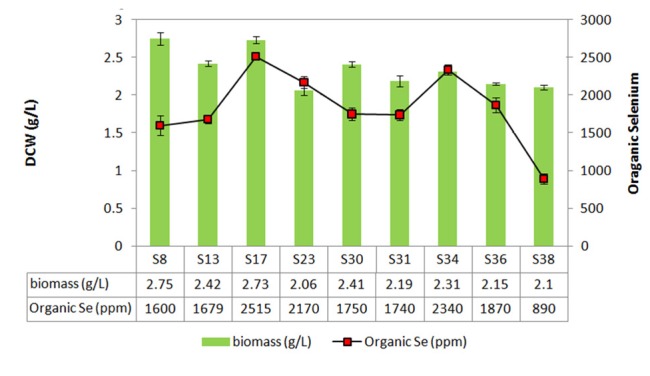
Screening of selenium tolerant yeast isolate for high yeast biomass and organic selenium production.

The 40 isolates from the previous study were cultured in SD broth medium containing different concentrations of sodium selenite (i.e., 5 mg/L, 15 mg/L and 25 mg/L) to separate species with high yeast biomass production. Biomass concentration of yeast isolates decreased; while selenium concentration increased from 5 mg/L to 25 mg/L (Figure 1). This decrease is due to selenium toxicity. One-way ANOVA used in order to find any significant variation between yeast biomass production in 3 groups (The mean difference is significant at the 0.05 level). After incubation, only nine yeast cells (S8, S13, S17, S23, S30, S31, S34, S36 and S38) were gained to acceptable biomass (above 2.5 g/L DCW in 25 mg/L selenium) (Figure 2). Yeast cells can elevate resistance to toxic metals and therefore the yeast cells are able to obtain new metabolic capacities while exposed to the selection pressure.^23^ Although the yeast cells can grow in medium with selenium, their ability to selenium biotransformation must be examined.

### Screening for selenium biotransformation

The best selected isolates from previous step were grown in SD broth containing 25 mg/L of sodium selenite. After incubation organic selenium content was measured. Organic selenium accumulation of the S8, S13, S17, S23, S30, S31, S34, S36 and S38 isolates were 1600, 1679, 2515, 2170, 1750, 1740, 2340, 1870 and 890 ppm respectively. The S17 isolate demonstrated greatest organic selenium (2515 ppm) and biomass creation (2.73 g/L) in comparison to other isolates (Figure 2). S17 and S34 isolates have given high biomass and best organic selenium content. The S8 isolate with the high biomass production, had low organic selenium content. On the other hand, the S23 isolate had high organic selenium content with low biomass production. Two isolates with the high resistant to selenium and best selenium biotransformation (S17 and S34) were selected for further characterization. The results of sequencing the ITS region and the LSU rRNA gene D1/D2 domains (data not shown) were indicated S17 and S34 isolates must be strains of *S. cerevisiae*.

### The relationship between growth and biotransformation

**Figure 3:**
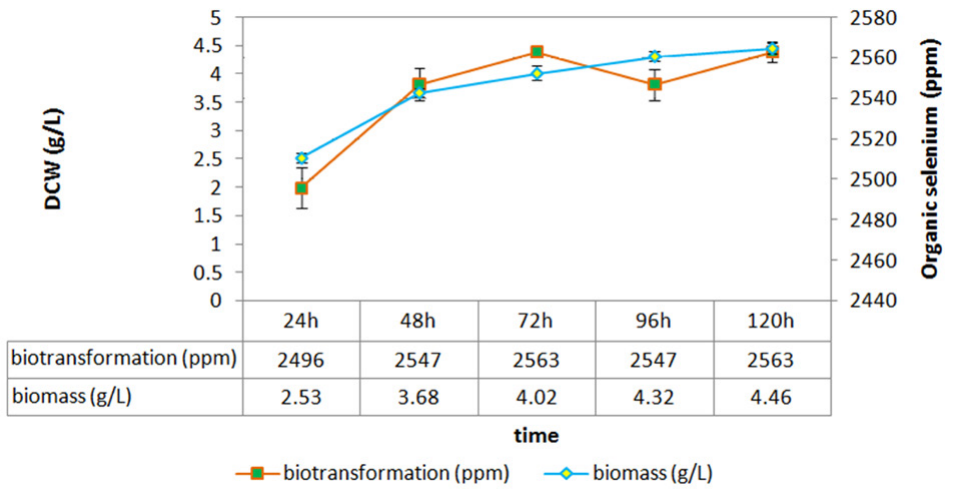
The relationship between biomass and selenium biotransformation in S17 isolate.

Finally, S17 isolate was grown in presence of selenium. Biomass and selenium biotransformation measurement at different times showed a direct relationship between biomass growth and selenium biotransformation in the first 72 hours (Figure 3).

Previous studies had shown the selenium amount that could be entered into yeast cells is between 500 to 3000 ppm.^4^ Marinescu and Stoicescu showed organic selenium production in the range of 300 to 2200 ppm in yeast cells by supplementation of the medium with 30 to180 μg/mL sodium selenite.^6^ Maximum biotransformation of selenium (2718 ppm) was acquired with synthetic medium by Rajashree and Muthukumar.^4^ Suhajda et al reported Seleno-yeast with 1200 to 1400 µg selenium per gram dried yeast.^9^

## Conclusion

Even though selenium species are formed by *Lactobacillus* and *Saccharomyces*; but selenomethionine (the major selenium-containing amino acid) is synthesized in *S. cerevisiae* yeast.^23^ On the other hand *S. cerevisiae* is one of the most known probiotics in the food industry and human nutrition.^24^ It is interesting to know that organic selenium has high bioavailability and low toxicity compared with inorganic selenium.^4^ Therefore, finding yeast strains resistant to selenium can be very helpful. In this study, the results showed strain (S17) capable of producing 2515 ppm organic selenium in presence of 25 mg/L of selenium, which is higher than the other isolates used in this work. This selenium biotransformation was performed only with screening, without manipulating the media.

## Ethical Issues

Not applicable.

## Conflicts of Interest

The authors have declared that no conflict of interest exists.

## Acknowledgments

This study is proposed and approved by the school of Pharmacy, Shahid Beheshti University of Medical Sciences, Tehran, Iran.
